# Multi-country cross-sectional study of colonization with multidrug-resistant organisms: protocol and methods for the Antibiotic Resistance in Communities and Hospitals (ARCH) studies

**DOI:** 10.1186/s12889-021-11451-y

**Published:** 2021-07-16

**Authors:** Aditya Sharma, Ulzii-Orishikh Luvsansharav, Prabasaj Paul, Joseph D. Lutgring, Douglas R. Call, Sylvia Omulo, Kayla Laserson, Rafael Araos, Jose M. Munita, Jennifer Verani, Fahmida Chowdhury, Syeda Mah-E Muneer, Andres Espinosa-Bode, Brooke Ramay, Celia Cordon-Rosales, C. P. Girish Kumar, Tarun Bhatnagar, Neil Gupta, Benjamin Park, Rachel M. Smith

**Affiliations:** 1 Division of Healthcare Quality Promotion, U.S. Centers for Disease Control and Prevention, Office of the Director, 1600 Clifton Rd NE, MS H16-2, Atlanta, GA 30029 USA; 2 Paul G. Allen School for Global Animal Health, Washington State University, 240 SE Ott Road, Pullman, WA 99164 USA; 3 Instituto de Ciencias e Innovación en Medicina Universidad del Desarrollo, Av. Las Condes, 12461 Santiago, Chile; 4 Millennium Initiative for Collaborative Research on Bacterial Resistance (MICROB-R), Av. Las Condes, 12461 Santiago, Chile; 5 Division of Global Health Protection, KEMRI Complex, Kenya Office, Mbagathi road off Mbagathi Way, PO Box 606-00621, Nairobi, Kenya; 6 icddr, b, 68 Shaheed Tajuddin Ahmed Sarani, Dhaka, 1212 Bangladesh; 7 Division of Global Health Protection, Central America Region Office, Edificio Instituto de Investigación 2 (II-2), Interior Universidad Del Valle, 18 Avenida 11-37, Vista Hermosa 3, Zona 15, Guatemala City, Guatemala; 8 Center for Health Studies, Universidad del Valle de Guatemala, Guatemala City, Guatemala; 9 National Institute of Epidemiology, II Main Road, TNHB, Ayapakkam, Chennai, 600 077 India

**Keywords:** MDRO, Antimicrobial resistance, Global health security

## Abstract

**Background:**

Antimicrobial resistance is a global health emergency. Persons colonized with multidrug-resistant organisms (MDROs) are at risk for developing subsequent multidrug-resistant infections, as colonization represents an important precursor to invasive infection. Despite reports documenting the worldwide dissemination of MDROs, fundamental questions remain regarding the burden of resistance, metrics to measure prevalence, and determinants of spread. We describe a multi-site colonization survey protocol that aims to quantify the population-based prevalence and associated risk factors for colonization with high-threat MDROs among community dwelling participants and patients admitted to hospitals within a defined population-catchment area.

**Methods:**

Researchers in five countries (Bangladesh, Chile, Guatemala, Kenya, and India) will conduct a cross-sectional, population-based prevalence survey consisting of a risk factor questionnaire and collection of specimens to evaluate colonization with three high-threat MDROs: extended-spectrum cephalosporin-resistant Enterobacteriaceae (ESCrE), carbapenem-resistant Enterobacteriaceae (CRE), and methicillin-resistant *Staphylococcus aureus* (MRSA). Healthy adults residing in a household within the sampling area will be enrolled in addition to eligible hospitalized adults. Colonizing isolates of these MDROs will be compared by multilocus sequence typing (MLST) to routinely collected invasive clinical isolates, where available, to determine potential pathogenicity. A colonizing MDRO isolate will be categorized as potentially pathogenic if the MLST pattern of the colonizing isolate matches the MLST pattern of an invasive clinical isolate. The outcomes of this study will be estimates of the population-based prevalence of colonization with ESCrE, CRE, and MRSA; determination of the proportion of colonizing ESCrE, CRE, and MRSA with pathogenic characteristics based on MLST; identification of factors independently associated with ESCrE, CRE, and MRSA colonization; and creation an archive of ESCrE, CRE, and MRSA isolates for future study.

**Discussion:**

This is the first study to use a common protocol to evaluate population-based prevalence and risk factors associated with MDRO colonization among community-dwelling and hospitalized adults in multiple countries with diverse epidemiological conditions, including low- and middle-income settings. The results will be used to better describe the global epidemiology of MDROs and guide the development of mitigation strategies in both community and healthcare settings. These standardized baseline surveys can also inform future studies seeking to further characterize MDRO epidemiology globally.

## Background

Antimicrobial resistance is a significant threat to global health security with the potential to stymie effective treatment and prevention of infections caused by a wide range of microorganisms [1]. Infections by multidrug-resistant organisms (MDROs) such as extended-spectrum cephalosporin-resistant Enterobacteriaceae (ESCrE, also known as third-generation cephalosporine-resistant Enterobacteriaceae), carbapenem-resistant Enterobacteriaceae (CRE), and methicillin-resistant *Staphylococcus aureus* (MRSA) can lead to disability or mortality, prolonged illness, and incur greater treatment costs [2–5] than those caused by more susceptible organisms. Addressing MDROs in low- and middle-income countries is particularly challenging, as health systems in these settings may not be prepared to effectively detect and prevent spread [6].

Originally described as infections of hospitalized patients, MDROs have since been documented in communities worldwide [7–10]. Studies of healthy travelers returning to the United States or Europe from countries in sub-Saharan Africa and South Asia indicate that some travelers are colonized with ESCrE and CRE during their journeys even in the absence of exposure to healthcare facilities or to antibiotics, suggesting that there is considerable colonization pressure in communities in these regions [11–14]. First reported in hospitalized patients, incident MRSA infections have more recently been described among persons in the community across multiple countries [15–19]. High prevalence of MDROs in communities may facilitate importation of these bacteria into healthcare settings, where they may spread further and cause infections in hospitalized patients.

Colonization with MDROs such as ESCrE, CRE, and MRSA is asymptomatic but increases the risk of multidrug-resistant infections [20–23] and can result in the unrecognized spread of MDROs to non-colonized persons [24, 25]. Though both hospitalized patients and healthy individuals may be colonized by MDROs, it is unclear to what extent colonizing MDROs are similar to MDROs that cause invasive disease. Increasing availability of genetic sequencing data, such as multilocus sequence typing (MLST), permits comparison of pathogenic isolates to colonizing MDROs and assists in inferring potential invasiveness of colonizing MDRO isolates [26].

Effective surveillance of MDROs is essential to guide prevention programs. Contemporary efforts for tracking MDROs are focused on describing antimicrobial resistance of clinical isolates. In many countries this type of surveillance is aligned with the framework of the World Health Organization Global Antimicrobial Resistance Surveillance System (GLASS), which focuses on reporting antimicrobial resistance in clinical isolates from persons attending healthcare facilities [27]. However, focusing on MDROs isolated from clinical specimens alone may not fully describe the magnitude and proximate determinants of MDRO spread in surveillance populations due to the latency between MDRO colonization and subsequent infection. The limitations of current approaches for MDRO surveillance may hinder development of robust MDRO prevention and control strategies.

Population-based studies complement facility-based surveillance for a number of disease conditions including HIV, latent tuberculosis infection, and MRSA carriage, to yield more complete estimates of prevalence, improve understanding of associated risks, and track progress of prevention programs [28–32]. Methods used in population-based studies have the potential to be adapted to describe MDRO colonization. Consistent with this opportunity and the need to enhance understanding of MDRO spread, we herein describe a common protocol for a coordinated study in six sites to assess the population-based prevalence of and risk factors associated with colonization with three MDROs of public health importance: ESCrE, CRE, and MRSA. For each country, the objectives of our study are fourfold: 1) estimate the population-based prevalence of colonization with ESCrE, CRE, and MRSA, 2) determine the proportion of colonizing ESCrE, CRE, and MRSA with pathogenic characteristics, 3) identify factors (e.g., patient characteristics) independently associated with ESCrE, CRE, and MRSA colonization; and 4) create an archive of ESCrE, CRE, and MRSA isolates for future study.

## Methods/design

### Study sites

Geographic sites selected for participation must meet several criteria to participate in the study:

1. Must have a population of at least 10,000 individuals;
2. Must have, or be able to rapidly develop, a sampling frame for households;
3. Presence of at least one operational hospital that serves the population residing in the sampling frame (i.e., the sampling frame is within the catchment area of the hospital);
4. Must have access to a bacteriology laboratory, or network of laboratories, that can isolate bacteria from biological specimens, perform identification and antibiotic susceptibility testing of bacterial isolates, perform or refer bacterial isolates for multilocus sequence typing, and archive specimens and isolates for up to 5 years.

Six sites in five countries were selected: 1) Dhaka, Bangladesh; 2) Molina, Chile; 3) Quetzaltenango, Guatemala; 4) Asembo, Kenya; 5) Nairobi, Kenya; and 6) Chennai, India.

### Study population

There are two target populations for the study: 1) healthy adults in households, and 2) hospitalized adults.

Eligibility criteria for enrollment of healthy adults in households are the following:

- At least 18 years old;
- Without fever, diarrhea, or cough at the time of interview and specimen collection;
- Must have slept overnight in the household for at least 4 weeks.

Eligibility criteria for enrollment of hospitalized adults are the following:

- At least 18 years old;
- Without diarrhea or active gastrointestinal bleeding at the time of enrollment;
- Documented severe neutropenia, defined as absolute neutrophil count < 500 (swabs may carry a risk of infection in the setting of skin and mucosal barrier breakdown).

Populations residing in sub-regions could be excluded if operations are not deemed feasible (e.g., regions of insecurity, military zones). Additional target populations (e.g., healthy children less than 5 years old) may be enrolled based on interest, operational capacity, and funding of in-country partners.

### Sample size

The estimation for sample size of eligible healthy adults in the community is based on the following formula.

1 $$N=\frac{z^2r\left(1-r\right)f}{(dr)^2k}$$

N is the sample size;

z is the z-score for the desired confidence level of the estimate;r is an estimate of the proportion of MDRO colonization in the population;f is the design effect based on the sampling method;d is the relative precision of the estimate;k is the estimated response rate.

The sample size should account for the fact that sampling is done on a finite population in the geographic area of the study. Correcting the sample size N from [1] for a finite population is performed using the equation below:

2 $${N}_{FPC}=\frac{N}{1+\frac{N-1}{T}}$$

Here, N_FPC_ is the corrected sample size based on N (the sample size from eq. 1) and T (the size of the target population in the study area).

Without prior knowledge of the extent of colonization in the population, a reasonable estimate to use for the proportion of adult individuals colonized with an MDRO is 0.2 [33–36]. Based on a 95% confidence interval, design effect of 2 to account for clustering, response rate of 75%, a relative precision of 20%, and assumption that the size of the target population is at least 10,000, a sample size of 1000 healthy adults will be needed. Among these 1000 healthy adults, 750 would be expected to complete study participation procedures and therefore represent the effective sample size.

The effective sample size should also be evaluated for sufficient power to identify associations between MDRO colonization and risk factors. For a logistic regression model consisting of 45 predictor variables, significance level of 0.05, and an estimate that the model explains 4% of the variance in MDRO colonization, an effective sample size of 750 would have a power of 0.82.

A similar approach will be used to generate a target sample size for the hospitalized population in the study area. The estimation for the number of inpatients to be sampled is based on the eq. 1, with application of the finite population correction described in eq. 2. For the hospitalized population, N_FPC_ is the corrected sample size of adult inpatients based on N (the sample size of adult inpatients from eq. 1) and T (the total number of adult inpatients in the study area). Both N and T should be based on an average daily census over 1 month or longer.

For example, assuming a colonization proportion of 0.2 among hospitalized adults, a 95% confidence interval, design effect of 2, response rate of 75%, a relative precision of 20%, and assumption that the size of the hospitalized adult population is least 2000, a sample size of 678 hospitalized adults will be needed. A sample of 678 inpatients would have an effective enrollment of 509 inpatients. Additionally, for a logistic regression model consisting of 15 independent variables, significance level of 0.05, and an estimate that the model explains 4% of the variance in MDRO colonization, an effective sample of 509 adult inpatients would have a power of 0.85.

### Sampling design

#### Sampling of healthy adults

At study sites where simple random sampling is logistically not feasible, cluster sampling will be the preferred method for the community study. Where necessary, multiple stages will be used.. At the first stage, the number of clusters allocated to sampling units will be based on probability proportional to size sampling, in which a cluster is any well-defined geographic area of similar population size such as enumeration areas or blocks. In the second stage, households will be mapped and listed in each of the selected clusters; a fixed number of households will then be selected by simple random sampling. Finally, one individual will be randomly selected for study participation among all eligible adults in the household.

#### Sampling for hospitalized adults

Stratification with proportional allocation is the preferred method for the hospital study. The total number of hospitalized persons to be sampled in a particular hospital will be based on the proportion of hospitalized persons in the study area (defined as the geographic area represented by the community sampling frame) represented by the hospital. Simple random sampling will be used to select and enroll hospitalized patients on a daily basis until the target sample size is reached. Oversampling may be considered for clinical areas of interest such as intensive care units or for certain categories of admitted patients such as those admitted for more than two calendar days.

### Study implementation

#### Study duration

The study will run until the sample size is achieved and laboratory testing is completed. Total study duration will depend on the study team’s size, geographical distribution of the participants, and the capacity of the laboratory where the testing is performed. We anticipate the estimated time needed to complete the study will range from 12 -- 24 months.

#### Enrollment of healthy adults in communities

Study staff will approach households selected from the sampling frame and will ask if the head-of-household (or household representative) is willing to participate in the study. If so, then study staff will request informed consent from the head-of-household or household representative to collect information about the household. Following documented written informed consent by the head-of-household, study staff will administer a questionnaire to capture household characteristics.

Next, from a list of all adult household members, study staff will randomly select one individual who meets eligibility criteria for further participation. Informed consent will be requested from the selected individual; if the individual declines or does not meet eligibility criteria, then an alternate will be randomly selected. After informed consent has been obtained, study staff will then administer a questionnaire to capture demographic and exposure history, including recent healthcare utilization, prior antibiotic use, occupational exposures, animal contact, access to safe water and sanitation facilities, and recent food sources.

#### Enrollment of hospitalized adults

Study staff will approach patients selected from the hospitals in the study area. Informed consent will be requested from hospital individuals who meet eligibility criteria. An adult caretaker must be present to provide informed consent for unconscious or sedated patients.

After informed consent has been obtained, study staff will collect data from medical records or patient interview on a standardized instrument. These data include demographic characteristics and relevant exposure history, including duration of hospitalization prior to enrollment, ward type and bed number on the day of the study, history of procedures, utilization of invasive medical devices, systemic antibiotic exposures, and results of microbiological tests. All information will be recorded by the study staff.

#### Collection of stool specimens

Enrolled participants will be asked to provide a stool specimen. At the household-level, study staff will give participants a stool collection kit to collect a fresh stool specimen as well as items to keep stool cold while awaiting pick up by study staff. Hospitalized patients will be given a stool collection kit or bed pan if bedridden. Study staff will also provide instructions for minimizing the risk of contamination by urine, toilet surfaces, and toilet water. Study staff will deliver stool specimens to the laboratory within 12 h of collection to the study laboratory.

In lieu of stools samples, certain enrolled participants may opt to have a rectal swab taken by trained study staff. Rectal swabs will be done via commercial kit using elution swabs (e.g. Copan Diagnostics Eswabs™) and will be obtained by inserting a sterile swab into the rectum, rotating then removing and placing into Liquid Amies transport media and kept at 4 °C until plating.

#### Collection of nasal swabs

At select sites detecting MRSA colonization, study staff will collect a single nasal swab (e.g., Copan Diagnostics Eswabs™) from enrolled participants by inserting a sterile swab into the naris, rotating against the anterior nasal mucosa, removing the swab, and then repeating with the other naris. After collection, swabs will be placed into a Liquid Amies transport media and kept at 4 °C until plating. Samples will be brought to the laboratory within 24 h of collection.

#### Laboratory procedures

Standard operating procedures (SOP) were developed for the transport and processing of study specimens and then adapted at each study site for use. On arrival at the study laboratory, all specimens will be reviewed for labeling, quantity of biological material, and length of time from specimen collection, sample integrity, and transport and storage under the correct conditions. Specimens that do not meet the criteria for acceptance (e.g., delayed transport time, leaking on arrival to lab, missing or incorrect label) will be rejected and documented accordingly.

Accepted specimens will be directly inoculated on separate culture media selective for each MDRO. For most sites this will be a commercially available brand of chromogenic agar selective for ESCrE, CRE, and MRSA (e.g., CHROMagar™, CHROMID™^)^; alternatives may include MacConkey agar with appropriate antibiotics for selection of these MDROs of interest. Stool specimens/rectal swabs will be inoculated on culture media selective for ESCrE and CRE. Nasal swab specimens will be inoculated on culture media selective for MRSA. Plates will be incubated in standard conditions for 18–24 h. If growth is present, up to three unique morphotypes from each plate with growth will be selected for archiving; these isolates will also undergo bacterial identification and antimicrobial susceptibility testing (e.g., via bioMerieux Vitek® 2), as well as PCR-based or whole genome MLST. Sites with technical expertise, supplies, and funding may inoculate specimens on additional selective media to detect colonization with other MDROs, such as colistin-resistant Enterobacteriaceae and carbapenem-resistant *Pseudomonas aeruginosa*.

During the study period, Enterobacteriaceae (e.g., *E. coli*, *K. pneumoniae*, *E. cloacae*) and *S. aureus* isolates from blood specimens at participating hospitals will be referred for MLST to generate a library of sequence types of locally detected infectious isolates in the study region.

### Pilot and monitoring

Before the formal study begins, study staff will undergo training on the protocol, standard operating procedures, and human subject’s protections related to the study. Worker safety issues will be addressed for collection, transport, analysis, handling, storage and disposal of biological specimens in accordance with local regulations.

A pilot involving at least 10 individuals from the community and 10 hospitalized patients will be performed at each site to identify and address any issues related to enrollment, interviews, specimen collection, specimen transport, and specimen analysis. Any issues identified during the pilot period will be addressed before initiating the formal study.

Monitoring will be performed on an ongoing basis to assess patient recruitment and to assess the quality and timeliness of collected study data. On-site monitoring will be performed by designated staff at least once every 2 weeks during the study period. The site supervisor will confirm that 1) the rights and well-being of human subjects are protected; 2) the consent process is followed per protocol; 3) the consent forms are appropriately completed; 4) the reported study data are accurate and complete; and 5) the participant identity is secured separately from the data; 6) the study conducted is in adherence with the currently approved protocol.

Laboratory quality control will be performed at the lab conducting the microbiology testing throughout the course of the study, with corrections to isolation, identification, and/or antimicrobial resistance detection processes if warranted. Manufacturer-recommended quality control strains will be plated alongside specimens obtained during the study to ensure quality of results. Logs for equipment used during the study will be kept to document correct operations of all laboratory procedures.

Every 2 weeks during the study, microbiology monitors will inspect all records kept by the laboratories for the study (e.g., quality control logs, specimen results), will ask questions regarding relevant laboratory processes and adherence to study SOPs, will observe the operations of the site, will review the results of testing of simulated specimens, and will debrief the team with findings and any needed actions. The microbiology monitors will assist laboratory staff to correct any deficiencies identified, including supporting the provision of necessary supplies and resources for the duration of the study. Specimens tested during periods of deficiency will be considered for disposal with a compensatory increase in enrollment of study participants.

During the study period, the laboratories will follow their routine SOPs for equipment maintenance and calibration. They will also adhere to their standard biosafety and biosecurity procedures applicable to isolation, identification and susceptibility testing of antimicrobial-resistant organisms.

### Data management

Within 1 week of enrollment, documents related to study participants will be completed, checked for quality, scanned, and securely archived. Physical copies of all forms will remain at the field site until the end of the survey. Documents with identifying data will be destroyed at the end of the study. Where feasible, electronic instruments (e.g., tablet computers) will be used for data entry.

In-country staff will transcribe paper-based questionnaire data and laboratory results into a study database; transcribed records will be de-identified. Study staff will use a standard reporting template to summarize participant enrollment, specimen collection and processing, and laboratory results for regular review by technical monitors. Electronic data will be stored on password-protected computers accessible only by the study team.

### Phenotypic definitions

1. ESCrE colonization is defined as isolation of Enterobacteriaceae non-susceptible to ceftazidime, ceftriaxone, or cefotaxime in a stool specimen collected from a study participant.
2. CRE colonization is defined as isolation of Enterobacteriaceae with acquired resistance to ertapenem, meropenem, doripenem, or imipenem in a stool specimen collected from a study participant.
3. MRSA colonization is defined as isolation of *Staphylococcus aureus* resistant to oxacillin or cefoxitin in a nasal swab specimen collected from a study participant.

Resistance and non-susceptibility will be interpreted based on breakpoints established by Clinical & Laboratory Standards Institute in the M100 | Performance Standards for Antimicrobial Susceptibility Testing document.

### Analysis plan

Descriptive analysis will be performed to describe the demographic characteristics of study participants and prevalence of risk factors. Risk factors associated with colonization will be assessed by using mixed-effects multivariate logistic regression models. Colonization will be represented as a binary variable indicating either presence or absence of MDRO. Risk factors will be constructed as continuous or categorical variables in accordance with the type of data captured. Additional models may be constructed to examine associations between risk factors and additional outcomes of interest such as colonization with specific MDRO species (e.g., carbapenem-resistant *Escherichia coli*).

Estimates of prevalence of MDRO colonization will be presented with 95% confidence intervals and will be adjusted for participation completion rates and sampling method; missing value imputation and sensitivity analysis will be performed where necessary.

At each study site, a colonizing isolate will be categorized as potentially pathogenic if the MLST pattern of the colonizing isolate matches the MLST pattern of a locally detected invasive isolate (defined as isolates growing from sterile sites such as blood only) obtained from participating hospitals or from public libraries of MLST patterns (e.g., PubMLST). For example, a colonizing isolate of carbapenem-resistant *K. pneumoniae* will be designated as potentially pathogenic if its MLST pattern is present in the library of MLSTs of *K. pneumoniae* isolates from blood specimens.

## Patient and public involvement

Patients and the public were not involved in the design of the study and will not be involved in study implementation.

### Ethics and dissemination

The protocol and subsequent amendments must receive ethical approval by appropriate national and local governments, academic bodies, and hospital administrators. The approval process must adhere to all local customs, standards, and regulations. All documents that are presented in a language other than English must be accompanied by a certificate from an independent observer confirming that the translated document is a locally appropriate translation of the English documents.

The results from the project will be summarized to in-country government bodies and health officials. Findings from the study will be disseminated in peer-reviewed journals and will also be presented at scientific conferences.

## Discussion

This protocol summarizes the rationale and approach of a study to determine the population-based prevalence of and associated risk factors for colonization with MDROs in multiple countries. This approach is complementary to conventional approaches of tracking antimicrobial resistance, which typically rely on characterization of clinical isolates from patient receiving healthcare. However, given the challenges of conducting surveillance with minimal bias (e.g., variable culturing practices and procedures), our study is positioned to generate critical data of the magnitude and proximate drivers of MDRO spread in diverse epidemiological settings. This study will also be valuable in generating data from a variety of low- and middle-income settings as often these areas have high rates of MDROs but generate little surveillance data to guide prevention and control efforts.

The strengths of the study include the utilization of existing platforms for population-based studies, simplified diagnostic procedures to detect MDRO colonization, enrollment of individuals in communities and hospitals from the same geographic setting, and implementation in multiple sites with diverse epidemiological characteristics to enhance understanding of MDRO spread. Categorization of colonizing isolates as potentially pathogenic based on MLST pattern will aid in estimating the human health consequence of MDRO colonization. Additionally, the archive of MDROs will provide opportunities for further characterization and analysis. In the future, sites that have generated baseline data can serve as a foundation for future studies looking at genomic markers of resistance, microbiome analyses and other work to better understand the global epidemiology of MDROs.

Given the observational study design this study will assess associations, not causal relationships between exposure and MDRO colonization. Inferences regarding the human health consequence of MDRO colonization are based on comparison of MLST of colonizing and infectious isolates. However, some MDROs with MLST not typically associated with pathogenic bacteria could cause infections; conversely, not all MDROs with an MLST similar to pathogenic bacteria will cause infections given host and environmental factors.

In summary, measuring the population-based prevalence of and risk factors associated with MDRO colonization is necessary to understand the magnitude of MDRO spread, prioritize areas for coordinated mitigation efforts, and potentially develop a metric to monitor success of prevention programs without relying on clinical infection data which are often biased towards capturing sicker patients who may have more resistant bacteria. The results from our study will add important information to existing surveillance approaches to track antimicrobial resistance and will provide insights on strategies to combat this global threat.

### CDC disclaimer

The findings and conclusions in this report are those of the authors and do not necessarily represent the official position of the US Centers for Disease Control and Prevention.

## Data Availability

Not applicable.

## Abbreviations

CRE: Carbapenem-resistant Enterobacteriaceae
ESCrE: Extended spectrum cephalosporin resistant Enterobacteriaceae
MDRO: Multi-drug resistant organism
MLST: Multi-locus sequence typing
MRSA: Methicillin resistant *Staphylococcus aureus*
SOP: Standardized operating procedure
